# Supplementary material for: Role and mechanism of NCAPD3 in promoting malignant behaviors in gastric cancer
Source: Front Pharmacol. 2024 Apr 22;15:1341039. doi: 10.3389/fphar.2024.1341039 (PMC11070777; doi:10.3389/fphar.2024.1341039)
Supplement: Supplementary file 11 [file DataSheet2.ZIP › GSEA/GO_CC/my_analysis.Gsea.1599461408869/GO_MEMBRANE_REGION.html]

Details for gene set GO\_MEMBRANE\_REGION[GSEA]

|  || Dataset | filtered\_dataset.sample\_info.cls#WT\_versus\_NCAPD3\_MUT |
| Phenotype | sample\_info.cls#WT\_versus\_NCAPD3\_MUT |
| Upregulated in class | NCAPD3\_MUT |
| GeneSet | GO\_MEMBRANE\_REGION |
| Enrichment Score (ES) | -0.3206528 |
| Normalized Enrichment Score (NES) | -1.583514 |
| Nominal p-value | 0.04221636 |
| FDR q-value | 0.13524634 |
| FWER p-Value | 0.779 |
Table: GSEA Results Summary

  

Fig 1: Enrichment plot: GO\_MEMBRANE\_REGION      
 Profile of the Running ES Score & Positions of GeneSet Members on the Rank Ordered List

  

| SYMBOL | TITLE | RANK IN GENE LIST | RANK METRIC SCORE | RUNNING ES | CORE ENRICHMENT || 1 | 659 | BMPR2 | 16 | 1.047 | 0.0659 | No |
| 2 | 3667 | IRS1 | 83 | 0.821 | 0.0790 | No |
| 3 | 538 | ATP7A | 305 | 0.588 | -0.0368 | No |
| 4 | 8737 | RIPK1 | 493 | 0.477 | -0.1363 | No |
| 5 | 203068 | TUBB | 736 | 0.358 | -0.2843 | No |
| 6 | 8772 | FADD | 742 | 0.355 | -0.2617 | No |
| 7 | 1605 | DAG1 | 823 | 0.304 | -0.2969 | Yes |
| 8 | 1984 | EIF5A | 846 | 0.259 | -0.2936 | Yes |
| 9 | 8837 | CFLAR | 865 | -0.278 | -0.2860 | Yes |
| 10 | 6385 | SDC4 | 871 | -0.282 | -0.2687 | Yes |
| 11 | 4973 | OLR1 | 944 | -0.366 | -0.2936 | Yes |
| 12 | 4864 | NPC1 | 964 | -0.384 | -0.2789 | Yes |
| 13 | 5621 | PRNP | 970 | -0.387 | -0.2539 | Yes |
| 14 | 91663 | MYADM | 1004 | -0.407 | -0.2476 | Yes |
| 15 | 7132 | TNFRSF1A | 1055 | -0.449 | -0.2504 | Yes |
| 16 | 1604 | CD55 | 1090 | -0.468 | -0.2404 | Yes |
| 17 | 857 | CAV1 | 1098 | -0.475 | -0.2102 | Yes |
| 18 | 5359 | PLSCR1 | 1146 | -0.511 | -0.2063 | Yes |
| 19 | 330 | BIRC3 | 1224 | -0.600 | -0.2175 | Yes |
| 20 | 1956 | EGFR | 1266 | -0.657 | -0.1984 | Yes |
| 21 | 54742 | LY6K | 1331 | -0.755 | -0.1887 | Yes |
| 22 | 2040 | STOM | 1389 | -0.988 | -0.1567 | Yes |
| 23 | 7462 | LAT2 | 1399 | -1.138 | -0.0791 | Yes |
| 24 | 493 | ATP2B4 | 1401 | -1.166 | 0.0065 | Yes |
Table: GSEA details [plain text format]

  

Fig 2: GO\_MEMBRANE\_REGION      
 Blue-Pink O' Gram in the Space of the Analyzed GeneSet

  

Fig 3: GO\_MEMBRANE\_REGION: Random ES distribution      
 Gene set null distribution of ES for **GO\_MEMBRANE\_REGION**
